# Opening the Blood-Brain Barrier and Improving the Efficacy of Temozolomide Treatments of Glioblastoma Using Pulsed, Focused Ultrasound with a Microbubble Contrast Agent

**DOI:** 10.1155/2018/6501508

**Published:** 2018-11-11

**Authors:** Qian Dong, Lin He, Linbo Chen, Qiongzhen Deng

**Affiliations:** ^1^Department of Cardiovascular Medicine, The First Affiliated Hospital of Chongqing Medical University, Chongqing, China; ^2^Department of Ultrasonography, The First Affiliated Hospital of Chongqing Medical University, Chongqing, China; ^3^Key Laboratory of Birth Defects and Reproductive Health of National Health and Family Planning Commission (Chongqing Population and family planning Science and Technology Research Institute), Chongqing, China

## Abstract

**Objective:**

To explore the effects of pulsed, focused, and microbubble contrast agent-enhanced ultrasonography (mCEUS) on blood-brain barrier (BBB) permeability and the efficacy temozolomide for glioblastoma.

**Methods:**

Wistar rats (n = 30) were divided into three groups (n = 10 per group) to determine optimal CUES conditions for achieving BBB permeability, as assessed by ultrastructure transmission electron microscopy (TEM) and western blot assays for the tight junction protein claudin-5. Optimized mCEUS effects on BBB permeability were subsequently confirmed with Evans blue staining (2 groups of 10 rats). The glioma cell line 9L was injected into the brain striatum of Wistar rats. After temozolomide chemotherapy, we detected glial fibrillary acidic protein (GFAP) levels in serum by enzyme-linked immunosorbent assay (ELISA) and in brain tissue by western blot, immunocytochemistry, and real-time quantitative polymerase chain reaction (qPCR).

**Results:**

BBB permeability was maximized with 1 ml/kg contrast agent mCEUS delivered via 10-min intermittent launches with a 400-ms interval. Evans blue staining confirmed BBB permeability following ultrasonic cavitation in the control group (P < 0.05). Following temozolomide chemotherapy, levels of the tumor marker GFAP were increased in the group with ultrasonic cavitation compared with the control group (P < 0.05).

**Conclusions:**

When rats were treated by mCEUS with intermittent launches (interval, 400 ms) and injected with 1 mg/kg contrast agent, BBB permeability was increased and temozolomide BBB penetration was enhanced, therapeutic enhancement for glioblastoma.

## 1. Introduction

The blood-brain barrier (BBB) is a major impediment to intracerebral diffusion of drugs used to treat brain cancer, reducing the efficacy of therapeutic drugs by preventing them from penetrating into the brain [1]. Pulsed, focused microbubble contrast agent-enhanced ultrasonography (mCEUS) is a technology that focused ultrasound treatment performing after intravenous microbubble contrast agent intravenous injection. Microbubble contrast agent media are gas-filled microbubbles with diameter usually around 2-5*μ*m. Microbubbles have a high degree of echogenicity that is the ability of an object to reflect ultrasound waves. What is more, a series of microbubble vibrations can lead to cavitation effect and the microflow [2–4]. These mechanical effects can increase BBB permeability and thus improve the efficacy of some drug treatments [5]. Consequently, mCEUS is regarded as a promising tool for delivering targeted drugs to the brain [6].

Among primary brain tumors, glioblastoma is the most malignant and has the worst clinical prognosis. It has aggressive and invasive growth characteristics, leading to short recurrence-free intervals, poor prognoses, and short survival times. Despite advances in surgical techniques, radiotherapy, and adjuvant chemotherapy, the quality of life and life expectancy of patients with gliomas have remained nearly unchanged for several years. Temozolomide has been proven to be the first-line chemotherapeutic drug chemotherapy drug for glioma treatment and can extend patient survival [7, 8].

Glial fibrillary acidic protein (GFAP) in serum is a diagnostic marker of brain glioma presence, with GFAP levels having been demonstrated to provide significant prognostic information [9, 10]. Several recent studies have indicated that GFAP may be useful as a broader biomarker, in addition to being a prognosticator, for patients with different types of solid tumors wherein increased expression of GFAP often marks tumor cells with a tendency to mature, signaling a better prognosis [11–13]. Ki-67 antigen, an antigen associated with cell proliferation, is expressed only in proliferating cells, and its detection level can be used to reliably evaluate proliferation activity of tumor cells. Studies have shown that the Ki67 proliferation index is closely related to the degree of glioma differentiation, infiltration, metastasis, and prognosis and is one of the important reference indicators for judging the prognosis of tumors [14].

A previous study showed that noninvasive focused ultrasound treatment enhanced delivery of temozolomide through the BBB such that the chemotherapeutic drug dosage could be increased specifically in the tumor region. Focused ultrasound-enhanced delivery of temozolomide significantly suppressed tumor growth and prolonged animal survival, suggesting that this approach may improve the future therapeutic outcome of brain tumor temozolomide chemotherapy [15]. We further studied the therapeutic effects of different mCEUS protocols. The aims of our study were twofold. In experiment 1, we sought to identify an optimal mCEUS protocol for achieving BBB permeability. BBB integrity was evaluated, in part, by assessing levels of claudin-5, a key protein of BBB endothelial cell tight junctions and important regulatory target in cerebrovascular endothelial permeability [16, 17]. In experiment 2, we examined whether the mCEUS treatment can augment the curative effect of temozolomide on parenchymal tumors and compare the curative effect of different protocols.

## 2. Materials and Methods

### 2.1. Experiment 1: CEUS Optimization Study for BBB Permeability

#### 2.1.1. Experiment 1A Animals

Thirty Wistar rats (200 ± 20 g; Huafukang Biotechnology Company, Beijing, China) were fed pellet feed for a week in cages before the experiment. The rats were divided equally into three groups: (a) non-mCEUS control group; (b) continuous launch of mCEUS [18]; and (c) intermittent mCEUS launches (400-ms interval) [18]. Before undergoing mCEUS, the rats were anesthetized by intraperitoneal injection of pentobarbital sodium (3%, 30 mg/kg). An acoustic window from the right eye to the vertex attachment of the right ear was prepared by shaving. To imitate the human condition of ultrasonic penetration, the rats' skull plates were removed and replaced by young adult human temporal bone pieces.

#### 2.1.2. Ultrasonography

Microbubble contrast suspension containing 8 *μ*l (45 *μ*g) of sulfur hexafluoride per ml (Third Military Medical University) was injected (1 ml/kg, both mCEUS groups) via the tail vein [18]. Continuous or intermittent mCUES was applied to each rat for a period of 10 min via a Philips IE33 (USA) ultrasonic diagnostic machine with an S5-1 probe (harmonic frequency, 1.7/3.3 MHz; mechanical index, 0.8 mI; and treatment time, 10 min) [19].

#### 2.1.3. Transmission Electron Microscopy (TEM)

At the time of the craniotomy, the rats' chests were opened and perfused with 100–150 ml normal saline. Once colorless flow was observed in the right atrium, brain tissue dissection was initiated. The brain tissues were fixed in glutaraldehyde (2.5%), dehydrated, embedded, and sectioned, after which the removed brain tissues were subjected to TEM.

#### 2.1.4. Western Blot Analysis

Total protein was extracted from a portion of the brain tissues using RIPA lysis buffer, separated by reducing SDS-PAGE (10% gels), and transferred to nitrocellulose membranes (Millipore, USA). The membranes were blocked with 5% BSA in Tris-buffered saline and Tween-20 (10 mM Tris, pH 7.5, 140 mM NaCl, 0.05% Tween-20) for 2 h at room temperature. A rabbit polyclonal antibody against claudin-5 (1:1000, Santa Cruz, USA) was used as the primary antibody, and horseradish peroxidase-conjugated goat anti-rabbit IgG (Sigma, USA) was used as the secondary antibody. Immunoreactive bands were detected using a SuperSignal West Dura Extended Duration Substrate kit (Thermo Scientific). The band intensity was calculated by ImageJ software.

#### 2.1.5. Experiment 1B: Evans Blue Staining to Detect the Permeability of the BBB

Wistar rats (n = 20) were divided into control and experimental groups (n = 10 for each). After anesthesia, Evans blue (2%, 50 mg/kg) was injected into the rats through the tail vein. The experimental group was treated with the optimized CUES protocol from the prior experiment. One hour later, they were perfused transcardially with 100–150 ml normal saline until the flow from the right atrium was colorless, after which the brain tissues were removed by craniotomy. The range of brain tissues stained by Evans blue was documented.

### 2.2. Experiment 2: Acoustic Cavitation Effects on BBB Permeability and Temozolomide Efficacy

#### 2.2.1. Animal Model

We injected the L9 glioma cell line into the brain striatum of 30 rats as described in detail previously [20]. To begin, we thawed the 9L cells, allowed them to recover in culture, and determined their cell viability. We then adjusted the density of the exponential-phase cells to 10^6^/20 *μ*l and inoculated them into the brain striatum using a stereotactic apparatus to induce glioma genesis.

#### 2.2.2. Temozolomide and mCUES Treatments

A cohort of 20 glioma model animals were divided into control and experimental groups (n = 10 each) and then treated with temozolomide (100 mg/kg; Jia Rui Biotechnology Company, Beijing, China) by daily intragastric administration for 5 d. A previous preclinical study reported that temozolomide was eliminated rapidly with a half-life of 1.2 h in rats (males and females) and an absolute oral availability as high as 96% [21].

After temozolomide delivery, rats in the experimental group were injected with contrast agent and mCUES was applied to the tumor location daily for 5 d, using our experimentally optimized protocol. The dose was selected based on correlation with the human dosing regimen and consistent with what has been typically applied in rodent glioma model [15, 22]. Control animals were injected with contrast agent but not subjected to mCUES.

#### 2.2.3. Enzyme-Linked Immunosorbent Assay (ELISA)

Samples of rat blood (0.5 ml) were collected from the angular vein and centrifuged. Serum GFAP levels were measured with a rat GFAP ELISA kit (Millipore, USA). All samples were run in duplicate and had an inter-/intra-assay variability < 10%.

#### 2.2.4. Tumor Volume and Inhibitory Rate

The animals were killed and brain tissues were removed. The volume of tumors in the control and test groups was measured and the tumor inhibition rate was calculated.

#### 2.2.5. Western Blot Analysis

Tumor tissues were analyzed by western blot as described above.

#### 2.2.6. Immunocytochemistry

Tumor tissues were fixed in paraformaldehyde (4%) for 20 min, embedded in paraffin, and cut into 5-*μ*m-thick sections with a microtome. Following heat-mediated antigen retrieval in citrate buffer, sections were permeabilized for 10 min. A rabbit polyclonal antibody against GFAP (1:1000, Abcam, USA) was used as the primary antibody, binding at 4°C overnight. Sections were washed three times in phosphate buffered saline and incubated with blocking solution, which included FITC-conjugated goat anti-rabbit IgG (RD, USA), at 37°C for 2 h. The sections were then washed three times in phosphate buffered saline and incubated with DAPI at 37°C for 5 min. Finally, the sections were observed by a laser confocal microscope.

#### 2.2.7. RNA Isolation and Real-Time qPCR

Total RNA was isolated from tumor tissues with RNAiso Plus (Takara, Japan) according to the manufacturer's instructions. First-strand complementary DNA was synthesized using oligo-dT primers and M-MLV reverse transcriptase (Takara). The qPCR was performed with SYBR green PCR Master Mix (Takara). The following primers were used: GFAP cDNA sense 5′-CGG GAG TCG GCG AGT TAC-3′ and antisense 3′-GGT GAT GCG GTT TTC TTC G-5′; *β*-actin cDNA sense: 5′-CCC ATC TAT GAG GGT TAC GC-3′ and antisense 3′-TTT AAT GTC ACG CAC GAT TTC-5′. PCR was performed using the following conditions: denaturing at 94°C for 20 s, annealing at 60°C for 30 s, and elongation at 72°C for 35 s. The mRNA levels of GFAP were normalized to *β*-actin.

### 2.3. Statistical Analysis

Data were reported as means ± SD and analyzed in SPSS 17.0. Comparative data were analyzed by multivariate analysis, and Student's t-test was used for paired data. A *P* value of < 0.05 was considered to be statistically significant.

## 3. Results

### 3.1. mCEUS Optimization for Improving BBB Permeability

**Figure 1:**
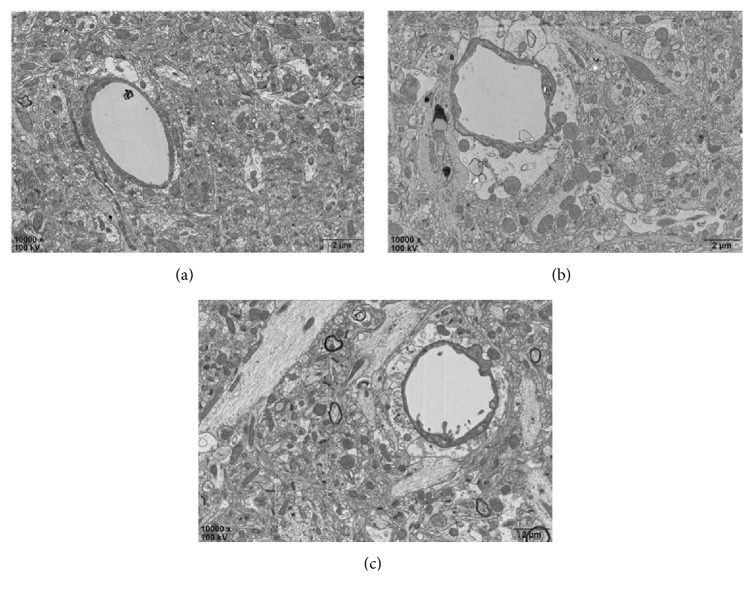
TEM of brain capillary tube ultrastructure. (a) Control group with basement membrane is thin and uniform and endothelial cells link closely. (b) Group with continuous launches and ultrasonic contrast agent (1 ml/kg). (c) Group with intermittent launches (interval, 400 ms) and ultrasonic contrast agent (1 ml/kg). In the latter two groups, note that the capillary lumen is irregular and the basement membrane is intact, while the thickness of the endothelial cells is uneven, outside the basal lamina, the astrocytes, and foot appear edematous, especially in (c).

**Figure 2:**
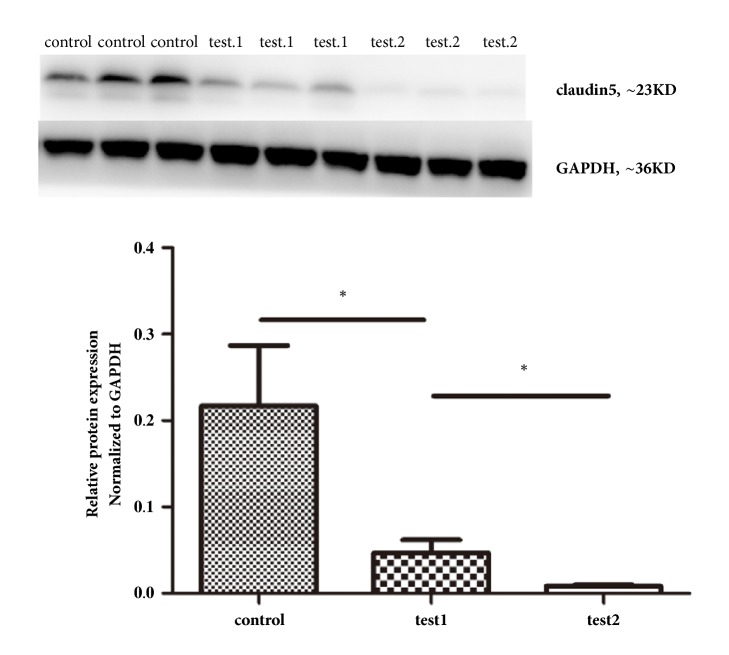
Western blot detection of claudin-5 (means ± SD, n = 3). test1: the group with continuous launches and ultrasonic contrast agent (1 ml/kg). test2: the group with intermittent launches (interval time of 400 ms) and ultrasonic contrast agent (1 ml/kg). Claudin-5 was reduced in the CEUS groups (test1 and test2) compared with the control group, and the reduction is especially pronounced in the intermittent group (test2).

TEM indicated that basement membranes of the capillary tube in the control group were thin and uniform with tightly linked endothelial cells. For the continuous mCEUS and intermittent mCEUS groups, the capillary lumen was irregular with intact basement membranes, but uneven endothelial cell thickness. Outside the basal lamina, the astrocytes and foot were edematous, especially for the intermittent mCEUS group (Figure 1). Western blot results showed that claudin-5 levels in the continuous mCEUS group and, to an even greater extent, the intermittent mCEUS group were reduced compared with the non-mCEUS control group. In other words, claudin-5 levels were most reduced in the intermittent mCEUS group (Figure 2). Studies have shown that the basis of the increased blood-brain barrier permeability is the reduced expression of claudin-5 mediated by caveolae [23]. So these results showed that intermittent mCEUS protocol (10 min of intermittent launches with 400-ms interval; harmonic frequency, 1.7/3.3 MHz; mechanical index, 0.8 mI; and treatment time, 10 min; 1 mg/kg contrast agent) was superior to the continuous mCEUS protocol for BBB permeabilization.

### 3.2. Evans Blue Staining Demonstrates mCUES-Induced BBB Permeability

**Figure 3:**
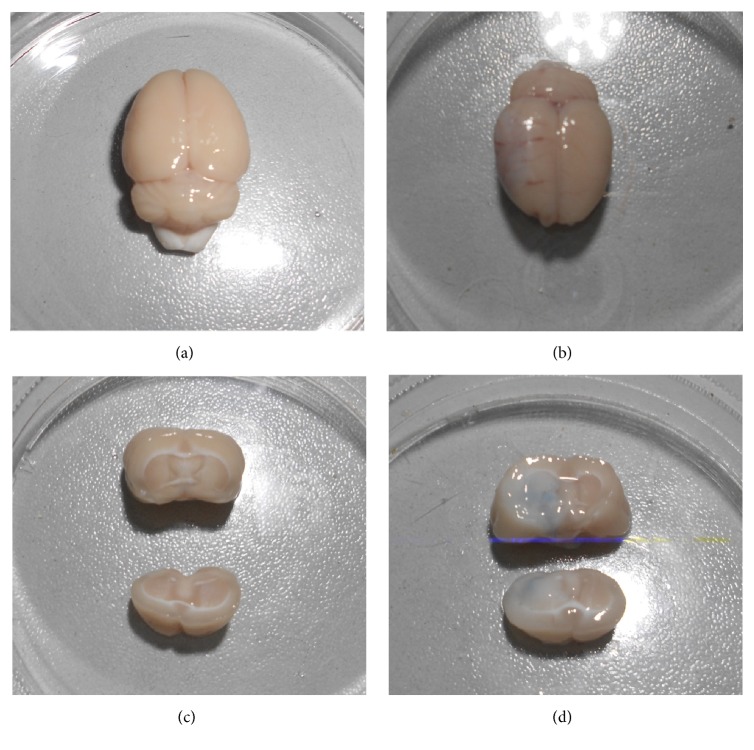
Detection of BBB permeability by Evans blue staining. No Evans blue staining was evident in the brain tissue of the control group (a). There was obvious Evans blue oozing from the brain tissue of the experimental group (b) consistent with the ultrasound direction.

No Evans blue staining was observed in the brain tissue of the control group, but there was obvious Evans blue oozing from the brain tissue of the test group, consistent with the ultrasound direction (Figure 3). The results showed that mCEUS cavitation produced BBB permeability.

### 3.3. Acoustic Cavitation Induced BBB Permeabilization Supports Temozolomide Efficacy

**Figure 4:**
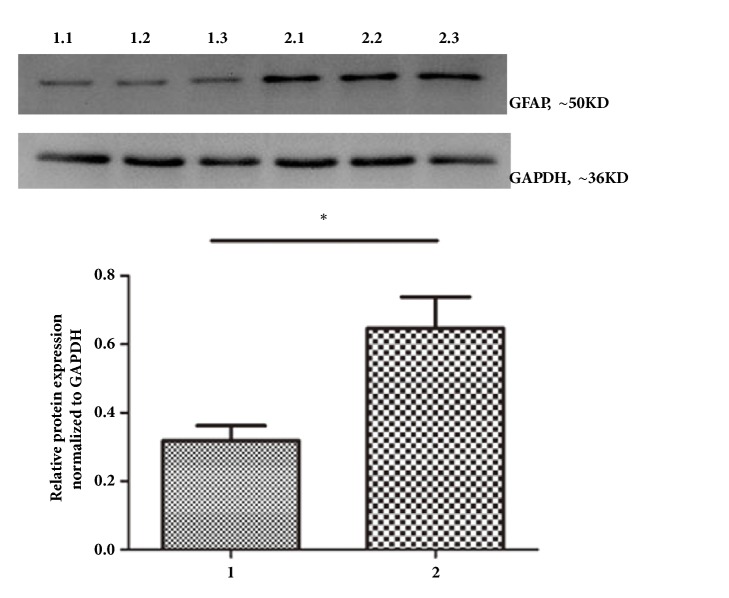
Western blot detection of GFAP (means ± SD, n = 3). GFAP expression in the tumor tissue was greater in test group than in the control group.

**Figure 5:**
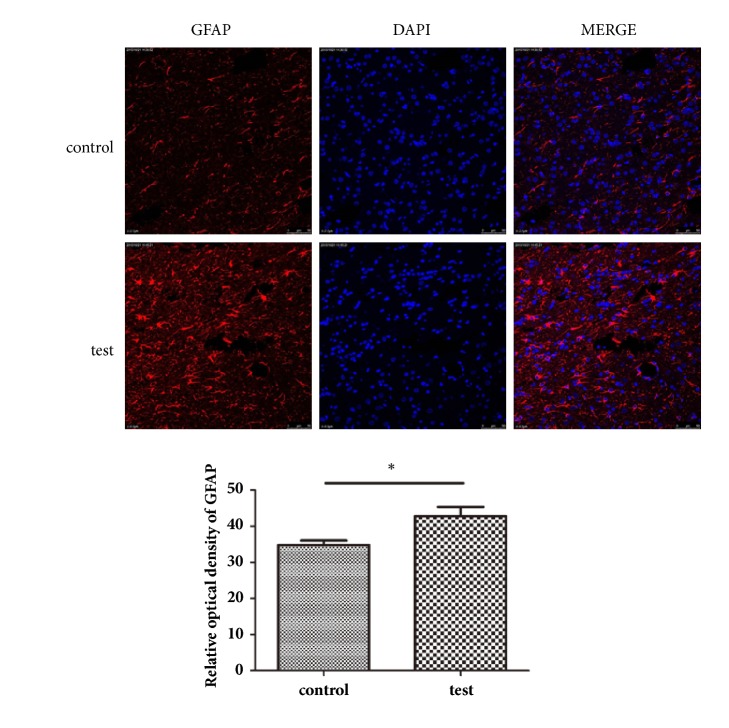
Detection of GFAP expression by immunocytochemistry analysis (means ± SD, n = 3). GFAP expression in the tumor tissue in the test group was increased compared with that in control group. GFAP expression was localized to the cytoplasm.

**Figure 6:**
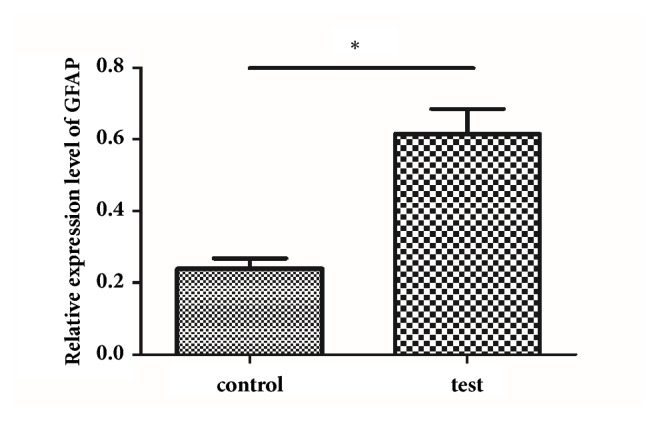
Detection of GFAP mRNA expression by real-time qPCR (means ± SD, n = 6). GFAP mRNA expression in tumor tissue in the test group was more abundant than that in the control group.

**Figure 7:**
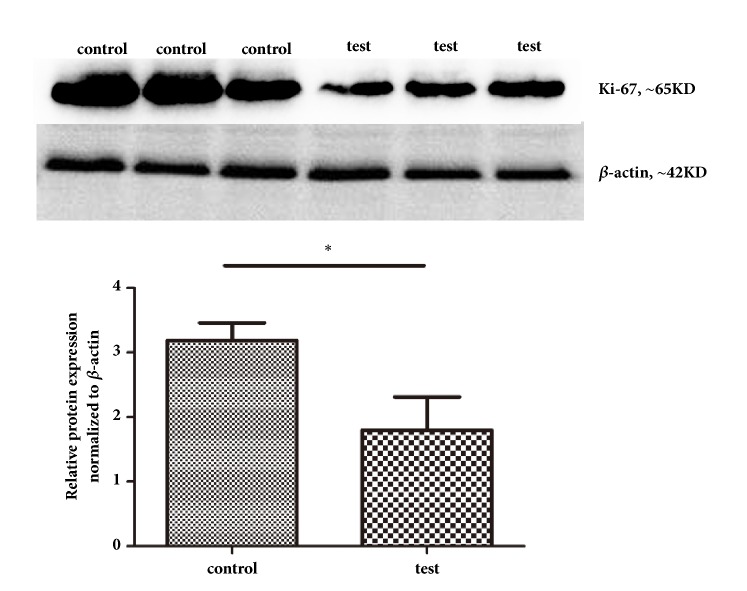
Western blot detection of Ki-67 (means ± SD, n = 3). Ki-67 expression in the tumor tissue was less in the test group than in the control group.

ELISAs showed higher serum GFAP levels in the experimental group than in the control group (Table 1). The tumor volume of the test group was lower than that of the control group, and the tumor inhibition rate was 36.2% (Table 2). Western blot and immunocytochemistry analyses showed greater GFAP protein expression in the tumor tissues of the test group than in the control group. GFAP expression was observed principally in the cytoplasm (Figures 4 and 5). GFAP mRNA expression in tumor tissues in the test group was also increased relative to that in the control group (Figure 6). Western blot analyses showed less Ki-67 protein expression in the tumor tissues of the test group than in the control group (Figure 7).

## 4. Data Sharing Statement

No additional unpublished data are available.

## 5. Discussion

In this study, we demonstrated the efficacy of pulsed, focused mCEUS, especially with our intermittent protocol (10 min of intermittent launches with 400-ms interval; harmonic frequency, 1.7/3.3 MHz; mechanical index, 0.8 mI; and treatment time, 10 min; 1 mg/kg contrast agent), for inducing BBB permeability using TEM, claudin-5 western blots, and Evans blue staining. We examined the effect of acoustic cavitation on temozolomide therapy efficacy by measuring tumor volume and testing GFAP and Ki-67 expression. Induction of permeability in temozolomide-treated glioma model rats was associated with reduction in tumor volume, increased GFAP expression, and reduced Ki-67 expression, suggesting that BBB permeabilization with this experimental technique can improve delivery of temozolomide to the brain. The similar study published in 2013 showed that noninvasive focused ultrasound treatment enhanced delivery of temozolomide through the BBB such that the chemotherapeutic drug dosage could be increased specifically in the tumor region [15], which is same as our study. But they did not test the effect of different protocols. We further studied the therapeutic effects of different mCEUS protocols.

The unique anatomical structure and physiological function of BBB endothelial cells relative to other endothelial cells underlie the high selectivity of the BBB. BBB endothelial cells have four noteworthy characteristics [24, 25]: (1) close connectivity with their surroundings; (2) relatively weak transmembrane transport activity; (3) intracellular mitochondria twice as numerous as in other endothelial cells suggestive of unusually active cellular metabolism; and (4) basic cell nutrients being provided mainly by specific transport molecules on the membrane. Meanwhile, on a tissue level, the BBB is unique with respect to its lacking contractile proteins and pinocytosis vesicles, its wealth of enzyme systems, and its negatively charged endothelial membrane surface. The specificity of the BBB preserves the stability of the intracranial environment, thereby protecting the normal metabolism and physiological functions of the brain. However, the BBB is a major obstacle to the delivery of therapeutic drugs to the nervous system, limiting drug treatments to small molecules [26]. Therefore, the BBB is a major obstacle to brain tumor chemotherapy.

Glioblastoma is a highly malignant glioma. It develops in the cortex, grows invasively and aggressively, violates the lobes, and affects the deep structure of the brain [27]. Because of these characteristics, patients with a glioblastoma often have a short recurrence-free interval, poor prognosis, and a short survival time. There is a grave need to explore a better therapeutic method to reduce the recrudescence rate and improve the quality of life of glioblastoma patients. Currently, the main therapeutic interventions for glioblastoma include surgical resection, radiation therapy, and chemotherapy [28]. Temozolomide is the most effective and commonly used chemotherapy drug in glioma treatment. It has good efficacy for relieving clinical symptoms, improving quality of life, and reducing postoperative adverse reactions. Therefore, improving the efficacy of temozolomide is vitally important for glioblastoma treatment.

It is noteworthy that the Evans blue staining observed in brains for which the BBB was subjected to intermittent mCEUS showed directionality consistent with the ultrasound direction. Evans blue combines with hemoglobin in the blood and does not penetrate into the extravascular space. Thus the penetration of Evans blue through blood vessel walls into the brain demonstrates capillary permeability changes [29].

Our observation of increased expression of GFAP, a marker of tumor cell maturation, and reduced expression of Ki-67, associated with cell proliferation, in mCEUS treated brains relative to non-mCEUS treated controls suggests that ultrasound under certain conditions can help temozolomide go through the BBB to better treat glioblastoma. Drug delivery appeared to be improved due to an acoustic cavitation effect, which is a physical effect caused by a series of microbubble vibrations in the microbubble contrast agent, causing expansion, contraction, and implosion. At the moment of microbubble implosion, energy is released rapidly in a small space, leading to a high temperature and pressure [2–4]. The shear stress produced by cavitation and the microflow produced by oscillation of microbubble cause local shock waves. These mechanical effects affect the mechanical sensitive proteins in endothelial cells, transforming the extracellular mechanical signals into intracellular biochemical signals [30], leading to a decrease in the integrity of the tight junctions of the blood-brain barrier [31, 32]. Caveolae are a cystic structure located on the surface of the cytoplasmic membrane and their main function is to mediate the endocytosis transport across cell membranes. Claudin-5 is tightly connected key protein which is highly expressed in all vascular endothelial cells in the brain. Studies have shown that the basis of the increased blood-brain barrier permeability is the reduced expression of claudin-5 mediated by caveolae [23, 33]. Thus it can be seen that the mechanical forces generated by focused ultrasound combined with microvesicles may affect the intercellular and paracellular pathways leading to a decline in the integrity of the tight junctions of the blood-brain barrier, leading to the opening of the blood-brain barrier. That would have an important impact for delivery of drugs across the BBB and thus enhance their curative effects.

Our study has some limitations. First, we tested a single contrast agent dose (1 ml/kg) and a single interval launch time (400 ms). Therefore, in further studies, it will be necessary to test different ultrasonic contrast agents and different interval launch times to potentially further optimize the protocol. Secondly, it will be important to explore the mechanisms of both ultrasonic acoustic cavitation and opening of the BBB, because they are unresolved and extremely complex.

In conclusion, pulsed, focused mCEUS can be used to permeabilize the BBB and augment the efficacy of temozolomide treatment for glioma. These results are relevant to improving pharmacotherapy efficacy in patients with glioblastoma.

## Figures and Tables

**Table 1:** ELISA-detected serum GFAP levels (means, n = 10).

Group	Mean GFAP level ± SD(*μ*g/ml)	*P*
Control	301.47 ± 7.85	< 0.05
Test	472.78 ± 4.95	

**Table 2:** The tumor volume (means, n = 10) and inhibitory rate.

Group	The tumor volume (mm^3^)	*P*	Inhibitory rate
Control	115.5 ± 20.2	< 0.05	36.2%
Test	73.6 ± 14.3		

## Data Availability

All the data used in the current study are within the article.
